# Impact of different treatment strategies on therapeutic efficacy and biomarkers in children with IVIG-resistant Kawasaki disease: a retrospective cohort study

**DOI:** 10.3389/fphar.2025.1602637

**Published:** 2025-06-25

**Authors:** Xin Zhou, Rui Dong, Xiaoling Wei, Hao Ding, Xiaochen Wang, Wei Wu, Guiyun Kang, Lei Li, Xiang Ma

**Affiliations:** ^1^ Department of Cardiology, Children’s Hospital Affiliated to Shandong University (Jinan Children’s Hospital), Jinan, China; ^2^ Pediatric Research Institute, Children’s Hospital Affiliated to Shandong University (Jinan Children’s Hospital), Jinan, China; ^3^ Department of Health Data Application and Management, Children’s Hospital Affiliated to Shandong University (Jinan Children’s Hospital), Jinan, China; ^4^ Department of Child Healthcare, Children’s Hospital Affiliated to Shandong University (Jinan Children’s Hospital), Jinan, China; ^5^ Community Management Department, Children’s Hospital Affiliated to Shandong University (Jinan Children’s Hospital), Jinan, China

**Keywords:** Kawasaki disease (KD), intravenous immunoglobulin (IVIG), glucocorticoids (GC), IVIG-resistant, retrospective study

## Abstract

**Background:**

Intravenous immunoglobulin (IVIG) is the standard first-line treatment for Kawasaki disease (KD), although 10%–20% of patients are resistant to initial IVIG therapy. This study investigates retreatment strategies and associated biomarkers in IVIG-resistant KD patients.

**Methods:**

This retrospective analysis included 68 IVIG-resistant KD patients from the Children’s Hospital Affiliated to Shandong University. Patients were categorized into three retreatment groups: glucocorticoids (GC) (Group A), IVIG retreatment (Group B), and combination therapy (Group C). Clinical characteristics, laboratory parameters, and therapeutic outcomes were compared, with multivariate logistic regression identifying biomarker correlations.

**Results:**

Despite significant differences in pre-treatment levels of C-reactive protein (CRP) and alanine aminotransferase (ALT) across groups, the combined overall response rate for all three groups following different retreatment strategies exceeded 98%. Multivariate logistic regression analysis identified pretreatment eosinophil percentage (EOS%) and albumin (ALB) levels as independent predictors of favorable outcomes, while elevated CRP was significantly associated with adverse outcomes. Furthermore, an increase in EOS% was observed after IVIG retreatment, suggesting a possible modulation of T helper 2 (Th2) immune responses by this intervention. Changes in coronary artery dilation further supported the potential benefits of GC monotherapy and combination therapy in mitigating acute vascular injury.

**Conclusion:**

Both GC and IVIG, either alone or in combination, are effective treatments for IVIG-resistant KD. EOS%, CRP, and ALB may serve as independent prognostic markers in children with IVIG-resistant KD, providing a foundation for personalized retreatment strategies.

## 1 Introduction

Kawasaki disease (KD) is an acute systemic vasculitis syndrome primarily affecting children under 5 years of age, clinically characterized by fever, polymorphous rash, bilateral conjunctival injection, oral mucosal changes, and erythema/desquamation of the extremities (Dhanrajani and Yeung, 2017; Kawasaki, 1967). As a significant pediatric condition, KD is most notably complicated by coronary artery lesions (CALs), which may lead to long-term cardiac sequelae and life-threatening outcomes (Gottlieb et al., 2023). Therefore, timely and effective intervention is crucial to prevent these severe consequences. The current standard of care for KD is intravenous immunoglobulin (IVIG), typically administered at a dose of 2 g/kg within the early stages of the illness to reduce systemic inflammation and lower the risk of coronary artery abnormalities (Terai and Shulman, 1997; Wang et al., 2020). However, despite its efficacy in the majority of cases, clinical reports indicate that approximately 10%–20% of patients demonstrate resistance to initial IVIG treatment (McCrindle et al., 2017). These patients face higher complication risks and require more aggressive intervention. While no universally accepted treatment protocol currently exists for IVIG-resistant KD, existing literature proposes various alternative or adjunctive strategies, including a second IVIG infusion, glucocorticoids (GC), infliximab, cyclosporine A, and other immunomodulators (Chang and Kuo, 2020; Rife and Gedalia, 2020).

This study screened out 68 IVIG-resistant KD patients from our center’s 1,107 KD patients and categorized them into three groups based on different retreatment regimens (GC, IVIG retreatment, and combination therapy). We retrospectively analyzed multiple clinical indicators before and after treatment to explore the clinical effects and biomarkers of retreatment options for IVIG-resistant KD patients. The goal is to explore more effective treatment combinations, providing valuable references for clinical practice.

## 2 Materials and methods

### 2.1 Study population

This retrospective study enrolled 68 IVIG-resistant KD patients admitted to the Children’s Hospital Affiliated to Shandong University between January 2021 and June 2024. Inclusion Criteria: met the diagnostic criteria for complete and incomplete KD according to the 2017 American Heart Association (AHA) guidelines for the diagnosis, treatment, and long-term follow-up of KD (McCrindle et al., 2017); continued fever ≥38°C 36 h after IVIG treatment or recurrence of fever within 2–7 days accompanied by at least one KD symptom, excluding secondary infections. Exclusion Criteria: missing data on initial IVIG therapy; presence of secondary bacterial infections; incomplete initial treatment records from other hospitals; recurrent KD cases; first IVIG treatment initiated more than 10 days after disease onset; failure to follow up as scheduled; use of GC or other medications within 1 month prior to KD onset; presence of comorbidities such as heart, lung, kidney, or endocrine system diseases.

### 2.2 Treatment protocols and grouping

**FIGURE 1:**
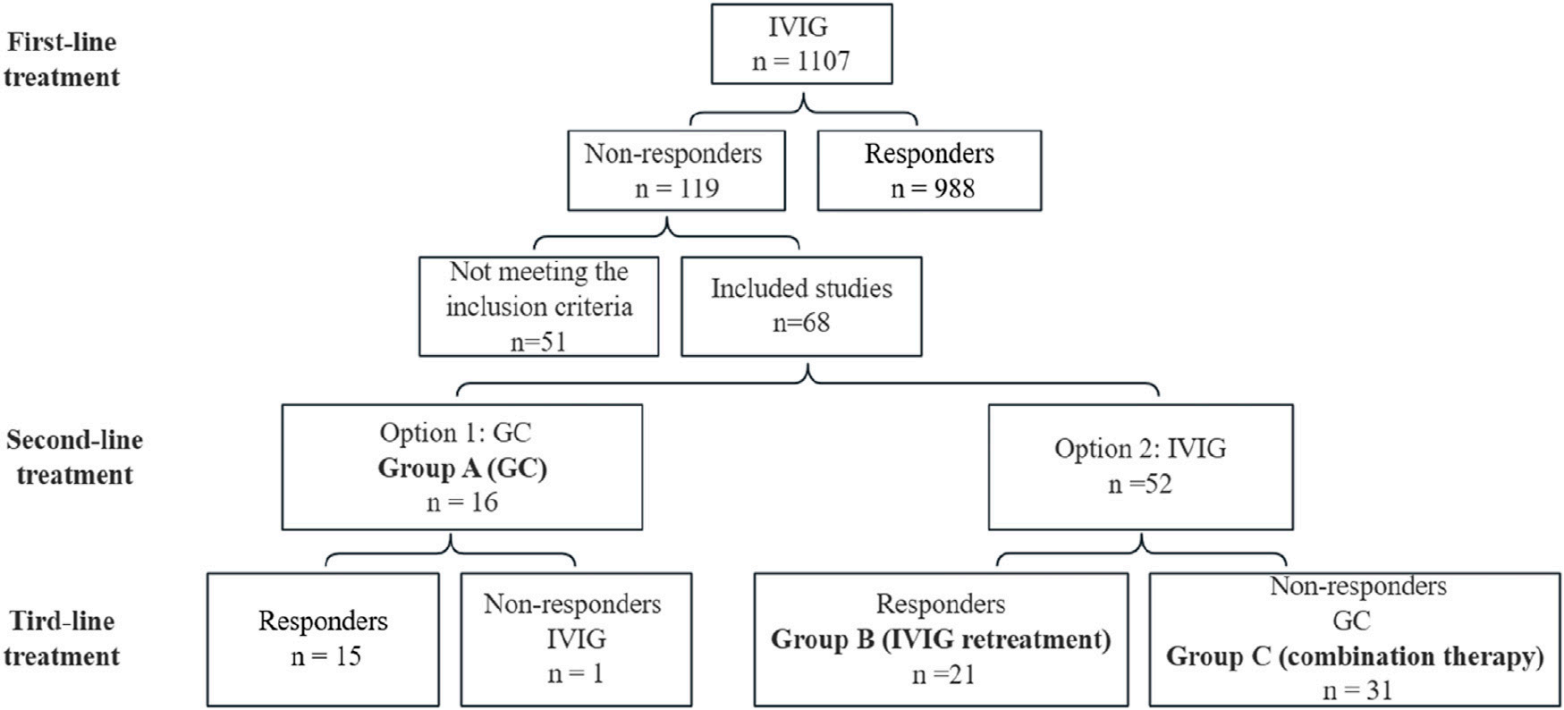
Protocol and results of the treatment in this study. IVIG, intravenous immunoglobulin; GC, glucocorticoids.

As shown in Figure 1, the clinical pathway for treating KD patients in our center is as follows:

Initial Treatment: All patients received IVIG at a dose of 2 g/kg, along with oral aspirin at 50 mg/(kgd), within 10 days of disease onset.

Retreatment Options for IVIG-Resistant Patients: For patients who did not respond to the initial IVIG treatment, retreatment options were selected in accordance with the 2017 AHA guidelines. Two main retreatment strategies were implemented:

Option 1: GC at a dose of 2 mg/(kgd) were recommended. If no response was observed, IVIG was administered as a second-line therapy. Patients receiving this regimen were categorized into Group A (GC).

Option 2: Repeated IVIG administration at a total dose of 2 g/kg was offered. If GC was required due to insufficient response, patients were classified into Group C (combination therapy). Those treated with IVIG alone were assigned to Group B (IVIG retreatment).

The final choice of retreatment regimen was made through shared decision-making between the attending physician and the patient’s family, considering clinical recommendations, parental preference, and financial conditions.

### 2.3 Observation indicators and evaluation criteria

Observation indicators: (1) Comparison of short-term efficacy and adverse reaction incidence among the three groups. (2) Observation of changes in white blood cell count (WBC), neutrophil count (NEUT), eosinophil ratio (EOS%), hemoglobin level (Hb), platelet count (PLT), C-reactive protein (CRP), erythrocyte sedimentation rate (ESR), procalcitonin (PCT), interleukin-6 (IL-6), tumor necrosis factor (TNF), alanine aminotransferase (ALT), albumin (ALB), age, weight, duration of fever before treatment, total fever course, etc., before and after treatment. (3) Patients were followed up for 3 months after treatment to observe the occurrence of coronary artery damage in the three groups.

Evaluation criteria: (1) Assess response effect 7 days after retreatment, calculate treatment efficacy rate, where effective means no recurrence of fever and significant disappearance of rash and other symptoms; ineffective means continued fever and KD symptoms, with echocardiography showing persistent coronary dilation. (2) Calculate total fever course (from fever onset to stable temperature for 48 h). (3) Monitor drug adverse reactions during treatment, measure body temperature, blood pressure, and blood glucose every 6 h, and perform echocardiography to confirm coronary dilation and arterial thrombosis. (4) Patients were followed up for 3 months to observe coronary artery damage at baseline (before treatment), and at 1 week, 4 weeks, 8 weeks, and 12 weeks after treatment. The observations primarily focused on coronary artery dilation, coronary artery aneurysms (CAA), and coronary artery stenosis.

### 2.4 Statistical analysis

Data processing and statistical analysis were performed using SPSS 20.0 software (IBM Corp.). Continuous variables were assessed for normality using the Kolmogorov-Smirnov test. Normally distributed data were expressed as mean ± standard deviation (SD), and intergroup comparisons were conducted using one-way analysis of variance (ANOVA), followed by pairwise comparisons with the Student-Newman-Keuls (SNK) *post hoc* test. Non-normally distributed variables were presented as medians (interquartile range), and differences among groups were analyzed using the Kruskal–Wallis test. Categorical variables were summarized as frequencies and percentages, and group comparisons were performed using the chi-square test. Univariate logistic regression analysis was initially used to identify potential covariates associated with treatment group effects. Variables showing statistical significance (P < 0.05) were then included in a multivariate logistic regression model to evaluate independent associations. A two-tailed *P* < 0.05 was considered statistically significant.

## 3 Results

### 3.1 General information

Among 1,107 KD patients treated at our center, 119 exhibited IVIG resistance. After excluding 33 cases with incomplete initial treatment records, 3 cases of delayed IVIG administration (>10 days post-onset), 2 recurrent KD cases, 5 cases with concurrent infections (3 bronchopneumonia, 2 sepsis), and 8 cases lost to follow-up, 68 eligible patients were enrolled. These patients were stratified into three groups: GC (Group A, *n* = 16), IVIG retreatment (Group B, *n* = 21), and combination therapy (Group C, *n* = 31).

### 3.2 Efficacy and adverse reactions

Groups A and B had a 100% efficacy rate, while Group C had a 96.8% efficacy rate, with one patient showing persistent coronary dilation. In Group A, 15 patients responded effectively to glucocorticoid therapy, with one patient requiring additional IVIG due to poor control of temperature and inflammatory markers.

Transient peripheral phlebitis occurred in 14 patients receiving repeated IVIG (Group B + C), resolving within 24 h of discontinuation. Five patients receiving GC treatment (Group A + C) exhibited increased neuroexcitability, manifested as crying and irritability, which resolved after discontinuation. No allergic reactions, hypertension, hyperglycemia, headache, vomiting, or other adverse reactions were reported in any of the groups.

### 3.3 Pre-treatment comparison

Comparative analysis of pre-treatment clinical parameters among the three groups (Table 1) revealed statistically significant differences in median CRP levels: Group C [51.49 (IQR: 30.00–89.52)] had significantly higher CRP levels than Group B [47.30 (33.08–65.26)] and Group A [30.54 (22.65–56.43)] (*F* = 8.47, *P* = 0.015). Group B exhibited a notably higher median ALT level [88.00 (53.00–177.00)] compared to Group C [37.00 (16.75–117.00)] and Group A [22.5 (16.00–105.5)] (*F* = 7.99, *P* = 0.018). No statistically significant differences were observed among the three groups for the remaining 13 parameters, including WBC count, NEUT count, EOS%, Hb level, and others (*P* > 0.05).

**TABLE 1:** Clinical parameters and statistical results of non-responders with KD before treatment across different therapeutic regimens.

Group	WBC (×10^9^/L)	NEUT (×10^9^/L)	EOS% (%)	Hb (g/L)	PLT (×10^9^/L)	CRP (mg/L)	ESR (mm/h)	PCT (ng/mL)	IL-6 (pg/mL)	TNF (pg/mL)	ALT (U/L)	ALB (g/L)	Weight (kg)	Age (y)	Pre-treatment fever duration (d)
A	14.65 ± 6.78	11.67 ± 5.75	2.4 (0.28, 5.6)	115.81 ± 7.53	386.06 ± 146.11	30.54 (22.65, 56.43)	51.13 ± 22.38	0.95 ± 1.80	328.29 ± 220.53	1.81 ± 0.54	22.5 (16.00, 105.5)	36.53 ± 3.13	16.52 (12.25, 19.25)	2.88 (1.69, 4.84)	6 (5.5, 7.5)
B	15.76 ± 4.50	10.91 ± 3.65	0.3 (0.1, 0.7)	109.53 ± 11.46	314.68 ± 135.12	47.30 (33.08, 65.26)	52.00 ± 28.35	1.98 ± 2.57	119.44 ± 135.27	1.99 ± 0.73	88.00 (53.00, 177.00)	34.32 ± 3.72	11.00 (8.00, 15.50)	2.08 (0.71, 3.46)	5 (4, 7)
C	15.36 ± 7.47	11.56 ± 5.56	0.3 (0.1, 2.1)	114.43 ± 11.31	389.30 ± 154.82	51.49 (30.00, 89.52)	56.93 ± 23.75	8.09 ± 20.00	564.35 ± 1667.85	3.12 ± 3.90	37.00 (16.75, 117.00)	34.45 ± 4.03	13.00 (11.00, 18.40)	3.00 (1.75, 4.5)	5 (4, 6)
*F*	5.94	9.25	14.43	1.84	1.57	8.47	2.76	1.71	4.01	2.44	7.99	1.94	5.72	3.74	3.72
*P*	0.15	0.10	0.07	0.17	0.22	0.015	0.3	0.19	0.22	0.33	0.018	0.15	0.057	0.154	0.16

WBC, white blood cell count; NEUT, neutrophil count; EOS%, eosinophil ratio; Hb, hemoglobin level; PLT, platelet count; CRP, C-reactive protein; ESR, erythrocyte sedimentation rate; PCT, procalcitonin; IL-6, interleukin-6; TNF, tumor necrosis factor; ALT, alanine aminotransferase; ALB, albumin; y, years; d, days.

Univariate logistic regression analysis of pretreatment variables between Groups A and B + C showed statistically significant differences in EOS% (*P* = 0.006; *β* = −0.38, OR = 0.68), CRP (*P* = 0.006; *β* = 0.03, OR = 1.029), and ALB (*P* = 0.001; *β* = −0.04, OR = 0.96). Fever duration before treatment approached significance (*P* = 0.059; *β* = −0.32, OR = 0.73). No other variables showed significant differences between groups.

Multivariate logistic regression analysis of variables with statistical significance in the univariate analysis (Table 2) confirmed the following associations: EOS% (*P* = 0.04; *β* = −0.367, OR = 0.693), CRP (*P* = 0.036; *β* = 0.26, OR = 1.026), ALB (*P* = 0.029; *β* = −0.036, OR = 0.964). Fever duration before treatment was not significant in multivariate analysis (*P* = 0.463; *β* = 0.202, OR = 1.224).

**TABLE 2:** Multivariate linear regression analysis between Group A and Group B + C.

SPSS	B	SE	Wals	df	Sig	EXP(B)	EXP(B)95%	95% CL
EOS%	−0.367	0.179	4.204	1	0.04	0.693	0.488	0.984
CRP	0.26	0.12	4.416	1	0.036	1.026	1.002	1.051
ALB	−0.036	0.17	4.763	1	0.029	0.964	0.934	1.91
pre-treatment fever duration	0.202	0.276	0.538	1	0.463	1.224	0.713	2.101
Constant response	1.98	1.631	1.474	1	0.225	7.242	1.00	1.01

EOS%, eosinophil ratio; CRP, C-reactive protein; ALB, albumin.

Univariate logistic regression analysis of variables (WBC count, Hb level, EOS%, CRP, ESR, IL-6, etc.) between Groups B and C revealed no statistically significant differences among these parameters.

### 3.4 Post-treatment comparison

Comparative analysis of clinical parameters among the three groups 5–7 days after treatment (Table 3) revealed statistically significant differences in the following metrics.

**TABLE 3:** Clinical parameters and statistical results of non-responders with KD after treatment across different therapeutic regimens.

Group	WBC (×10^9^/L)	NEUT (×10^9^/L)	EOS% (%)	Hb (g/L)	PLT (×10^9^/L)	CRP (mg/L)	ESR (mm/h)	PCT (ng/mL)	IL-6 (pg/mL)	TNF (pg/mL)	ALT (U/L)	ALB (g/L)	Total heat range
A	17.64 ± 11.41	9.39 ± 6.17	0.1 (0.08, 0.35)	113.94 ± 12.00	520.19 ± 119.36	2.57 (0.50, 4.16)	43.00 ± 17.44	0.15 ± 0.10	13.51 ± 16.89	0.62 ± 0.16	23 (17, 45)	32.25 ± 4.01	8.6 (8, 11.9)
B	12.08 ± 8.72	5.55 ± 6.83	2.1 (1.3, 2.6)	100.10 ± 16.69	653.38 ± 178.21	3.30 (0.50, 5.03)	60.75 ± 39.52	0.17 ± 0.08	398.85 ± 713.17	1.59 ± 0.34	23 (18, 35)	32.38 ± 3.23	9 (8, 11.5)
C	20.04 ± 10.35	13.39 ± 7.74	0.3 (0.1, 1.6)	106.68 ± 13.07	528.10 ± 161.65	3.30 (0.50, 5.84)	39.73 ± 21.28	0.44 ± 0.60	102.68 ± 105.06	1.25 ± 0.83	27 (22, 34)	32.82 ± 4.09	8.8 (7.25, 10.9)
*F*	3.85	7.66	9.04	4.41	4.75	3.53	1.26	1.59	1.09	1.29	0.67	0.10	0.36
*P*	0.03	0.001	0.0003	0.02	0.01	0.17	0.31	0.22	0.36	0.52	0.72	0.90	0.84

Data are expressed as the mean ± SD. KD, Kawasaki disease; WBC, white blood cell count; NEUT, neutrophil count; EOS%, eosinophil ratio; Hb, hemoglobin level; PLT, platelet count; CRP, C-reactive protein; ESR, erythrocyte sedimentation rate; PCT, procalcitonin; IL-6, interleukin-6; TNF, tumor necrosis factor; ALT, alanine aminotransferase; ALB, albumin.

The EOS% in Group B was significantly higher than that in the other two groups, with a median value of [2.1% (1.3%, 2.6%)], compared to [0.3% (0.1%, 1.6%)] in Group C and [0.1% (0.08%, 0.35%)] in Group A. Statistical analysis revealed a significant difference in EOS% among the groups (*F* = 9.04, *P* < 0.001). Additionally, both the WBC count and NEUT count were higher in Group C than in Groups A and B. The intergroup difference in WBC count was significant (*F* = 3.85, *P* = 0.03), while the difference in NEUT count was even more pronounced (*F* = 7.66, *P* = 0.0001). Regarding Hb levels, Group A exhibited significantly higher values than Groups B and C (*F* = 4.41, *P* = 0.02). Meanwhile, the PLT count in Group B was significantly higher than in the other two groups (*F* = 4.75, *P* = 0.01). No statistically significant differences were observed in other parameters such as CRP, ESR, and PCT among the three groups (*P* > 0.05).

Comparison of coronary artery dilation before and after treatment (Table 4) showed no significant differences among the three groups at baseline (*F* = 0.45, *P* = 0.80). At 1 week after treatment initiation, the number of patients exhibiting coronary artery dilation reached its peak across all groups. Specifically, for CAA type, Group A had 2 cases of mild and 1 case of moderate dilation; Group B had 3 cases of mild, 1 case of moderate, and 1 case of severe dilation; and Group C had 4 cases of mild and 3 cases of moderate dilation. Despite variations in the severity distribution, no statistically significant differences were observed between the groups at any time point in terms of the number or severity of CAA cases. With continued treatment over time—at 4 weeks, 8 weeks, and 12 weeks—the number of affected patients gradually declined. By 12 weeks post-treatment, the distribution of CAA types was as follows: Group A had 1 case of mild and 1 case of moderate dilation; Group B had 1 case of severe dilation; and Group C had 2 cases of mild and 1 case of moderate dilation.

**TABLE 4:** Comparison of coronary artery dilation pre- and post-treatment among different groups: case numbers and proportions.

Time	Group A (*n* = 16)	Group B (*n* = 21)	Group C (*n* = 31)	F	*P*
Pre-treatment	2 (12.50%)	3 (14.29%)	6 (19.35%)	0.45	0.80
1 w	3 (18.75%)	5 (23.81%)	7 (22.58%)	0.15	0.93
4 w	2 (12.50%)	2 (9.52%)	5 (16.13%)	0.50	0.78
8 w	1 (6.25%)	2 (9.52%)	3 (9.68%)	0.19	0.91
12 w	1 (6.25%)	1 (4.76%)	3 (9.68%)	0.49	0.78

w, week(s).

One week after treatment (Table 5), a borderline significant difference in coronary artery Z-scores was observed among the three groups (H = 6.00, P = 0.05). Group C had the lowest median Z-score [0.55 (0.23–1.01)], followed by Group A [0.80 (0.38–1.28)], and Group B had the highest values [0.92 (0.26–1.59)]. These findings suggest a potential early advantage of glucocorticoid-based regimens (particularly combination therapy) in reducing coronary artery dilation, although no significant differences were detected at later follow-up time points.

**TABLE 5:** Comparison of coronary artery Z value among different groups.

Time	Group A	Group B	Group C	H	*P*
Pre-treatment	0.90 (0.45, 1.26)	0.63 (0.32, 1.39)	0.48 (0.18, 0.98)	4.44	0.11
1 w	0.80 (0.38, 1.28)	0.92 (0.26, 1.59)	0.55 (0.23, 1.01)	6.00	0.05
4 w	0.84 (0.58, 1.27)	0.83 (0.29, 1.69)	0.72 (0.38, 1.24)	1.55	0.47
8 w	1.17 (0.40, 1.45)	0.56 (0.45, 1.27)	0.73 (0.29, 1.45)	0.96	0.63
12 w	1.00 (0.47, 1.39)	0.30 (0.13, 1.36)	0.91 (0.58, 1.28)	0.01	1.00
H	0.61	2.05	4.97		
*P*	0.90	0.56	0.17		

w, week(s).

## 4 Discussion

This retrospective study of 68 IVIG-resistant KD patients investigated the clinical efficacy and biomarker profiles of different retreatment regimens, including GC, IVIG retreatment, and combination therapy. Despite significant pretreatment disparities in CRP and ALT, all groups achieved high response rates. These baseline differences may reflect clinicians’ subjective assessments in treatment selection based on disease severity. Multivariate logistic regression identified pretreatment EOS% (OR = 1.42, 95% CI = 1.12–1.78) and ALB levels (OR = 1.24, 95% CI = 1.05–1.46) as independent predictors of favorable outcomes, while elevated CRP (OR = 1.68, 95% CI = 1.31–2.15) was significantly associated with adverse outcomes, suggesting these biomarkers could guide retreatment decisions. Post-treatment differences in laboratory parameters such as increased WBC count, NEUT count, and Hb level in GC-treated groups likely reflect glucocorticoid-induced hematopoietic modulation. Conversely, elevated EOS% and PLT count after IVIG retreatment may indicate T helper 2 (Th2) immune regulation and compensatory thrombopoiesis. The intergroup variations in coronary artery dilation further support GC’s potential advantage in mitigating acute vascular injury.

Studies have shown that IVIG non-responsive KD patients experience worsening conditions due to inadequate local inflammatory responses, emphasizing the importance of timely and effective anti-inflammatory treatment in KD management and complication prevention (Fukazawa et al., 2020). GC exerts a dual mechanism to effectively suppress systemic inflammation in KD: genomic pathways involve binding to cytoplasmic receptors, entering the nucleus, and directly regulating the expression of inflammation-related genes. For example, they inhibit the transcriptional activity of nuclear factor-κB (NF-κB) and activator protein-1 (AP-1), reducing the synthesis of proinflammatory cytokines (e.g., IL-1β, TNF-α) and adhesion molecules (e.g., VCAM-1), thereby blocking infiltration of neutrophils and monocytes into the vessel wall (Furusho et al., 1984). Non-genomic pathways rapidly inhibit inflammatory signaling within minutes, such as blocking phosphorylation of mitogen-activated protein kinase (MAPK) and extracellular signal-regulated kinase (ERK), reducing matrix metalloproteinase (MMP-9) release, preventing coronary artery elastic fiber rupture, and vascular remodeling (Glucocorticoid Emergency Application Consensus Expert Group, 2020). This dual mechanism not only shortens recovery times for fever and inflammatory markers (e.g., CRP, ESR) but also significantly reduces the risk of CAA by inhibiting endothelial damage. Multiple studies have demonstrated the safety and efficacy of GC. For instance, a meta-analysis of global data indicated that GC combined with IVIG shortened inflammation recovery time without increasing CAA risk (Zhu et al., 2012); further clinical research confirmed that GC combined with IVIG significantly reduced the incidence of coronary artery abnormalities in high-risk KD patients (Corticosteroids Added to Initial Intravenous Immunoglobulin Treatment for the Ae R et al., 2020). GC combined with IVIG has become a recommended second-line treatment for IVIG non-responsive or high-risk KD patients, supported by several high-quality studies (Huang et al., 2023).

Early identification of high-risk KD patients who do not respond to IVIG is crucial for improving prognosis. A relevant guideline (Expert Committee of Advanced Training for Pediatrician et al., 2022) summarizes the factors significantly associated with IVIG non-responsiveness and the risk of CAA: A Kobayashi score ≥5 points, determined by weighted scoring of six indicators including serum sodium ≤133 mmol/L, AST ≥100 IU/L, and neutrophil count ≥80%, achieves a sensitivity of 86% and specificity of 67% (Shin et al., 2017); infants aged <0.5 years exhibit a 43% incidence of CAA due to immature immune systems and delayed diagnosis (Corticosteroids Added to Initial Intravenous Immunoglobulin Treatment for the Ae R et al., 2020; Li et al., 2016); delayed treatment, defined as initiating IVIG therapy >10 days after onset, increases the risk of CAA by 9-fold (OR = 9.12, 95% CI = 7.63–10.90) (Çakan et al., 2016). Barman et al. (2024) further identified additional high-risk factors—Kawasaki disease shock syndrome (KDSS): approximately 60% of KDSS patients are resistant to IVIG, with a 62% CAA incidence (Kanegaye et al., 2009); macrophage activation syndrome (MAS): 41.7% of KD patients with MAS develop CAA, necessitating early intensive immunosuppressive treatment (Pilania et al., 2021); baseline coronary Z-score ≥2.5: initial echocardiograms showing abnormal Z-scores were associated with an 81% CAA incidence during follow-up, indicating the need for combined glucocorticoid treatment (Dominguez et al., 2012; Miyata et al., 2018). In this study, CRP and ALB were identified as independent risk factors in the local population. Elevated CRP aligns with the logic of the Kobayashi score warning system, while low ALB levels reflect ongoing vascular leakage and inflammation.

EOS% showed significant differences in pre-treatment multivariate logistic regression analysis and post-treatment group comparisons. This phenomenon might reflect the regulatory role of Th2 immune responses. A study by Tsai et al. (2021) on 6,310 febrile children indicated that EOS% > 1.5% is the strongest independent predictor of KD (sensitivity 82%, specificity 76%), carrying more weight than other inflammatory markers (e.g., ALT, CRP), suggesting EOS% as a supplementary indicator for identifying high-risk patients early. Kuo’s review further elucidates the biological significance of EOS%: eosinophils secrete anti-inflammatory mediators (e.g., IL-4, IL-13) that inhibit pro-inflammatory cytokines (e.g., TNF-α, IL-6), alleviating vascular endothelial damage and potentially protecting against CALs (Kuo, 2023). However, in the IVIG non-responsive subgroup, persistent elevation of EOS% (>2.0%) was significantly associated with abnormal coronary Z-scores (Z ≥ 2.5), showing a 3.5-fold higher CAL incidence compared to normal controls, suggesting that excessive Th2 responses may disrupt immune homeostasis and weaken the immunomodulatory effects of IVIG (Kuo, 2023). Although EOS% has not been incorporated into mainstream high-risk scoring systems (e.g., Kobayashi score), its independent predictive value in the local population aligns with previous mechanistic studies. Future studies should validate the optimal threshold of EOS% and its role in guiding individualized treatments, particularly in IVIG non-responsive high-risk populations.

## 5 Conclusion

This study proposes EOS%, CRP, and ALB as independent prognostic markers in children with IVIG-resistant KD, providing a foundation for personalized retreatment strategies. Patients with elevated baseline CRP and hypoalbuminemia may benefit from combination therapy (IVIG + GC), while those with high EOS% and low CRP levels could be prioritized for glucocorticoid monotherapy. The limitations of this retrospective study include the exclusion of 51 cases due to not meeting inclusion criteria, as well as relatively small sample sizes in three subgroups and the combined groups. These factors may affect the statistical power and generalizability of the findings. Therefore, prospective multicenter trials with larger cohorts are warranted to validate the predictive utility of these biomarkers and further explore their biological mechanisms, such as the association between EOS% and Th2 immune phenotypes.

## Data Availability

The original contributions presented in the study are included in the article/supplementary material, further inquiries can be directed to the corresponding authors.
